# Quantitative multi-parameter mapping of R1, PD^*^, MT, and R2^*^ at 3T: a multi-center validation

**DOI:** 10.3389/fnins.2013.00095

**Published:** 2013-06-10

**Authors:** Nikolaus Weiskopf, John Suckling, Guy Williams, Marta M. Correia, Becky Inkster, Roger Tait, Cinly Ooi, Edward T. Bullmore, Antoine Lutti

**Affiliations:** ^1^Wellcome Trust Centre for Neuroimaging, UCL Institute of Neurology, University College LondonLondon, UK; ^2^Department of Psychiatry, University of CambridgeCambridge, UK; ^3^Behavioural and Clinical Neuroscience Institute, University of CambridgeCambridge, UK; ^4^Cambridgeshire and Peterborough NHS Foundation TrustCambridge, UK; ^5^Department of Clinical Neuroscience, Wolfson Brain Imaging Centre, University of CambridgeCambridge, UK; ^6^MRC Cognition and Brain Sciences UnitCambridge, UK; ^7^GlaxoSmithKline, Clinical Unit Cambridge, Addenbrooke's HospitalCambridge, UK; ^8^Laboratoire de recherche en neuroimagerie, Département des neurosciences cliniques, CHUV, University of LausanneLausanne, Switzerland

**Keywords:** multi-center, T1, PD, MT, T2^*^, 3T, MPM, qMRI

## Abstract

Multi-center studies using magnetic resonance imaging facilitate studying small effect sizes, global population variance and rare diseases. The reliability and sensitivity of these multi-center studies crucially depend on the comparability of the data generated at different sites and time points. The level of inter-site comparability is still controversial for conventional anatomical T1-weighted MRI data. Quantitative multi-parameter mapping (MPM) was designed to provide MR parameter measures that are comparable across sites and time points, i.e., 1 mm high-resolution maps of the longitudinal relaxation rate (R1 = 1/T1), effective proton density (PD^*^), magnetization transfer saturation (MT) and effective transverse relaxation rate (R2^*^ = 1/T2^*^). MPM was validated at 3T for use in multi-center studies by scanning five volunteers at three different sites. We determined the inter-site bias, inter-site and intra-site coefficient of variation (CoV) for typical morphometric measures [i.e., gray matter (GM) probability maps used in voxel-based morphometry] and the four quantitative parameters. The inter-site bias and CoV were smaller than 3.1 and 8%, respectively, except for the inter-site CoV of R2^*^ (<20%). The GM probability maps based on the MT parameter maps had a 14% higher inter-site reproducibility than maps based on conventional T1-weighted images. The low inter-site bias and variance in the parameters and derived GM probability maps confirm the high comparability of the quantitative maps across sites and time points. The reliability, short acquisition time, high resolution and the detailed insights into the brain microstructure provided by MPM makes it an efficient tool for multi-center imaging studies.

## Introduction

Multi-center studies using magnetic resonance imaging (MRI) facilitate the detection of small effects, detailed estimation of neuroanatomical population variance and investigation of rare diseases. For example, recent multi-center studies identified reliable markers for Alzheimer's disease (Kloppel et al., 2008a,b), distributed anatomical differences in autism spectrum disorder (Ecker, 2012) or the relationship of inter-individual differences in character traits to anatomical differences (Schilling et al., 2013), which would have been difficult or impossible in a single-site setting.

However, the reliability and sensitivity of multi-center studies crucially depend on the comparability of structural MRI data generated at the different sites (Tofts and Collins, 2012). The level of inter-site comparability is still controversial for conventional MRI data. Some studies demonstrated systematic inter-site differences in structural T1-weighted images, which biased morphometric analyses (Focke et al., 2011), whereas other studies argued that typical pathology-related differences can be detected reliably by, for example, adjusting for potential inter-site differences (Pardoe et al., 2008; Stonnington et al., 2008; Suckling et al., 2012).

Quantitative anatomical MRI (qMRI) aims to overcome the inter-site bias issue, since it is specifically designed to provide absolute measures and thus data that are comparable across sites and time points (Tofts, 2003). Although various anatomical qMRI methods were developed (Tofts, 2003), there are only few studies that validated them in multiple centers. Deoni et al. (2008) validated the use of quantitative mapping of the longitudinal and the transverse relaxation time (T1 and T2) in a multi-center study at 1.5T. They demonstrated a high comparability between sites and reproducibility within a single site in scan-rescan experiments (<10% deviation). A magnetization transfer ratio (MTR) imaging protocol was optimized and validated in a multi-center study on multiple sclerosis at 1.5T (Barker et al., 2005; Ropele et al., 2005; Tofts et al., 2006). Careful alignment of imaging protocols and post-processing achieved a high comparability between sites [<4% deviation, (Ropele et al., 2005)]. The transverse relaxation time (T2) was quantified in a multi-center study on Alzheimer's disease and compared across sites at 1.5T (Bauer et al., 2010). Significant inter-site bias of up to 20% was observed, obscuring pathological changes.

These multi-center studies were performed at rather low resolutions (1.2–8.7 mm^3^), a field strength of 1.5T and investigated only few quantitative parameters. Spatial coverage was usually small and often did not allow for whole-brain imaging.

Recently, a comprehensive quantitative multi-parameter mapping (MPM) approach was developed at 3T (Helms et al., 2008a, 2009; Weiskopf et al., 2011), which provides high resolution maps of the longitudinal relaxation rate (R1 = 1/T1), effective proton density (PD^*^), magnetization transfer saturation (MT) and effective transverse relaxation rate (R2^*^ = 1/T2^*^). Whole-brain maps are acquired with 1 mm^3^ isotropic resolution in a clinically feasible time of approximately 24 mins. The multiple parameter maps and the high resolution allow for a detailed assessment of the white matter (WM) and gray matter (GM) tissue microstructure (Draganski et al., 2011; Dick et al., 2012; Sereno et al., 2012). MPM was used to study a wide range of different tissue changes in e.g., healthy aging (Draganski et al., 2011) or prosopagnosia (Garrido et al., 2009). The high sensitivity and specificity of the approach also improves segmentation of subcortical structures in morphometric studies (Helms et al., 2009). An advanced MPM approach using a higher isotropic resolution of 800 μm even allowed for mapping of cortical myelination and parcellation of brain areas (Dick et al., 2012; Sereno et al., 2012).

We validated the MPM approach at 3T for use in multi-center studies. The inter-site variance across three sites and intra-site variance were determined for typical morphometric measures (i.e., GM probability maps) and the four quantitative parameters. We also compared MPM to conventional T1-weighted (T1w) imaging using FLASH (fast low angle shot) imaging.

## Methods

### Participants and centers

The same five healthy volunteers [2 males, age 24.2 ± 1.6 yrs (mean ± *SD*)] were scanned at these three sites within 12 weeks: (1) Wellcome Trust Centre for Neuroimaging, London; (2) Medical Research Council Cognition and Brain Sciences Unit and (3) Wolfson Brain Imaging Centre, Cambridge (in the following called WTCN, MRC CBSU and WBIC, respectively). The study received ethical approval by the Cambridge Psychology Research Ethics Committee (ref: 2012.17) and all scanning sites obtained local approvals and written informed consent was obtained before scanning. This study was one of several pilot studies conducted to demonstrate the feasibility of the imaging approach used by the NeuroScience in Psychiatry Network (NSPN), which addresses how psychiatric disorders are related to abnormal maturation of brain systems.

### Data acquisition

All scans were acquired on 3T whole body MRI systems (Magnetom TIM Trio, Siemens Healthcare, Erlangen, Germany; VB17 software version) operated with the standard 32-channel radio-frequency (RF) receive head coil and RF body coil for transmission. The MPM comprised three multi-echo 3D FLASH (fast low angle shot) scans, one RF transmit field map and one static magnetic (B0) field map scan (Weiskopf et al., 2011). The MPM acquisition and post-processing were developed and optimized in previous studies and are briefly described here for convenience (Helms et al., 2008a,b, 2009, 2011; Lutti et al., 2010, 2012; Weiskopf et al., 2011).

Three different multi-echo FLASH scans were acquired with predominant T1-, PD-, and MT-weighting by appropriate choice of the repetition time (TR) and the flip angle α: TR/α = 18.7 ms/20° for the T1w scan and 23.7 ms/6° for the PDw and the MTw scans. MT-weighting was achieved by applying an off-resonance Gaussian-shaped RF pulse (4 ms duration, 220° nominal flip angle, 2 kHz frequency offset from water resonance) prior to the excitation. Multiple gradient echoes were acquired with alternating readout polarity at six equidistant echo times (TE) between 2.2 and 14.7 ms for the T1w and MTw acquisitions and at 8 equidistant TE between 2.2 ms and 19.7 ms for the PDw acquisition. Other acquisition parameters were: 1 mm isotropic resolution, 176 sagittal partitions, field of view (FOV) = 256 × 240 mm, matrix = 256 × 240 ×176, parallel imaging using GRAPPA factor 2 in phase-encoding (PE) direction, 6/8 partial Fourier in partition direction, non-selective RF excitation, readout bandwidth BW = 425 Hz/pixel, RF spoiling phase increment = 50°, total acquisition time ~19 min.

The design of the protocol took into account the following criteria. The primary goal was to acquire all three FLASH whole brain images with 1 mm resolution within ca. 20 min. The short acquisition time was achieved by combining GRAPPA parallel imaging with Partial Fourier acquisition. The moderate 2× GRAPPA acceleration avoided deterioration of image quality due to a poor geometry factor (Pruessmann et al., 1999). The echo train length was limited to ca. 20 ms to trade off emerging R2^*^ contrast against susceptibility-induced signal losses, while it allowed for determining R2^*^ and averaging of images for high SNR (Helms and Dechent, 2009). It also allowed for a high readout bandwidth (425 Hz/pixel) to minimize off-resonance and chemical shift artifacts. To achieve the same TR for the MTw and PDw scans, only 6 echoes were acquired for the MTw scan, to accommodate the 4 ms long off-resonance RF pulse. The 2 kHz frequency offset of the MT saturation pulse was chosen so that direct saturation effects were reduced and stability was increased (Helms et al., 2008b). The flip angle of the off-resonance pulse was titrated to keep the specific absorption rate (SAR) below ca. 75% of the normal mode SAR limit. The T1w scan was acquired with a shorter TR and fewer echoes to increase the signal-to-noise ratio (SNR) per time unit. The flip angles of the acquisitions were optimized using a semi-empirical approach, in order to maximize SNR while limiting bias due to imperfect RF spoiling (Yarnykh, 2010; Helms et al., 2011).

Maps of the local RF transmit field were measured and estimated from a 3D EPI acquisition of spin and stimulated echoes (SE and STE) with different refocusing flip angles (Lutti et al., 2010, 2012). Imaging parameters were: 4 mm isotropic resolution, matrix = 64 × 48 × 48 and FOV = 256 mm × 192 mm × 192 mm along readout × PE × partition direction, parallel imaging using GRAPPA factor 2 × 2 in PE and partition direction, TE_SE_/TE_STE_/TM (mixing time)/TR = 37.06/37.06/31.2/500 ms, acquisition time 3 min. The flip angles of the SE/STE refocusing pulses were decreased from 230°/115° to 130°/65° and in steps of 10°/5°.

To correct the 3D EPI RF transmit field maps for geometric distortion and off-resonance effects, a map of the static magnetic field (B0) was acquired with the following parameters (Lutti et al., 2010, 2012): 2D double-echo FLASH sequence with 64 axial slices, slice thickness = 2 mm, inter-slice gap = 1 mm, TR = 1020 ms, TE1/TE2 = 10/12.46 ms, α = 90°, matrix = 64 × 64, FOV = 192 × 192 mm, left-right PE direction, BW = 260 Hz/pixel, flow compensation, acquisition time ~2 min.

### Estimation of parameter maps

All data analyses and processing were performed in Matlab (The MathWorks Inc., Natick, MA, USA) using SPM8 (www.fil.ion.ucl.ac.uk/spm) and custom-made Matlab tools. Arithmetic mean T1w, PDw and MTw images were calculated from the first 6 multi-echo acquisitions (with the shortest TE), in order to increase the SNR. The resulting three mean images were used to calculate the parameter maps of the MT saturation, the apparent longitudinal relaxation rate R1 and the signal amplitude using previously developed models describing the image intensity of FLASH scans (Helms et al., 2008a,b; Weiskopf et al., 2011). The signal amplitude maps are proportional to PD but not corrected for RF receive field inhomogeneities and R2^*^ related effects (Helms et al., 2008a). The effective transverse relaxation rate R2^*^ was estimated from the logarithm of the signal intensities (from the 8 PDw multi-echo images) at different echo times using a linear regression.

Quantitative R1 maps were determined from the apparent R1 maps by correcting for local RF transmit field inhomogeneities and imperfect RF spoiling using the approach described by (Preibisch and Deichmann, 2009), which was adapted to the FLASH acquisition parameters used here. RF transmit field maps were calculated from the 3D EPI acquisition and corrected for off-resonance effects as described in (Lutti et al., 2012).

Effective PD^*^ maps were estimated from the signal amplitude maps by adjusting for global and local receive sensitivity differences. Since the local receive sensitivity can be described as a multiplicative factor between the signal amplitude and actual PD maps and receive sensitivity profiles are smooth, the UNICORT post-processing approach can be used for correction (Weiskopf et al., 2011). UNICORT is based on the new unified segmentation approach implemented in SPM8 that combines image registration, tissue classification, and multiplicative bias correction in a single generative model and optimizes its log-likelihood objective function (Ashburner and Friston, 2005). Since the global mean PD cannot be estimated accurately with this post-processing approach, we calibrated the mean WM PD value to 69 percent units [p.u.; (Tofts, 2003)]. Note that we did not correct for R2^*^ dependent signal decay by extrapolating the signal to *TE* = 0 but used the averaged multi-echo FLASH data with an effective TE = 8.45 ms. Because these resulting PD estimates still partly depended on R2^*^, we called this parameter the effective proton density (PD^*^) in line with previous reports (Lin et al., 1997).

The semi-quantitative MT saturation parameter is relatively robust against differences in relaxation times and RF transmit and receive field inhomogeneities—unlike the conventional MT ratio, which is affected by R1 and RF transmit field variations (Helms et al., 2008b, 2010). Additionally, small residual higher order dependencies of the MT saturation on the local RF transmit field were corrected using a semi-empirical approach, resulting in a corrected MT saturation value used in the further processing: $MT_{\text{corrected}}=\frac{MT_{\text{uncorrected}}\cdot (1-0.4)}{\left(1-0.4\cdot RF_{\text{local}}\right)}.MT_{\text{uncorrected}}$ is the original MT value and *RF*_local_ the relative local flip angle compared to the nominal flip angle (derivation and details of this correction will be reported elsewhere).

### Inter-site and intra-site variation

The R1, PD^*^, MT, R2^*^ maps, mean PDw and mean MTw from the three different sites were registered to the mean T1w image for each volunteer and interpolated using 4th order B-splines. The registered MT maps and mean T1w images were partitioned into cerebrospinal fluid (CSF), GM and WM using unified segmentation (Ashburner and Friston, 2005). T1w images are commonly used for brain segmentation but MT maps were shown to improve the segmentation of subcortical areas (Helms et al., 2009; Tardif et al., 2009). A brain mask was defined as all voxels whose sum of CSF, GM and WM probability exceeded 90%.

The inter-site coefficient of variation (CoV) for each parameter and weighted image was calculated by dividing the standard deviation (*SD*, normalized by the *N*−1 sample size to avoid bias) by the mean across the three sites (CoV = *SD*/Mean) for each volunteer independently. Mean and CoV maps were generated for visual assessment.

The mean inter-site CoV was determined for four different regions-of-interest (ROIs): head of the caudate nucleus, genu of the corpus callosum, GM and WM. The first two ROIs were manually defined within the head of the caudate nucleus and genu of the corpus callosum (bilateral). The MTw images were used for the delineation of these two ROIs, because they were not used as an outcome measure in this study, which minimized any potential bias or circularity in the following analyses. The other two ROIs described the whole-brain GM and WM and were defined as all voxels with a probability of GM and WM tissue over 99% as determined by the unified segmentation of the MT maps. The high probability threshold was used to reduce partial volume effects. To formally assess whether the four different parameter maps showed a lower inter-site CoV than the T1w image, a repeated measures ANOVA (SPSS Statistics 17.0; IBM Corp., NY) was performed with the within-subject factors ROI (4 levels) and image type (parameter map/T1w image) separately for each parameter map (significance threshold of *p* < 0.05). *Post-hoc* paired *t*-tests were conducted in case of a significant main effect of image type. We did not correct for multiple comparisons due to comparison with the four MPM parameters, since we regarded them as separate studies/experiments.

The systematic inter-site bias was assessed as the percent difference from the mean across all three sites and volunteers. The percent bias was averaged across the GM and WM ROIs for the R1, MT, R2^*^ parameters and T1w image. The measure for the PD^*^ map was averaged across the GM ROI only, since the WM PD^*^ value was calibrated to 69 p.u. in the post-processing and thus the bias might have been underestimated.

The intra-site CoV was calculated based on the *SD* and mean across all voxels within an ROI for each volunteer and site. The intra-site variation was estimated from the *SD* of the signal between voxels within the ROI [similar to previous studies (Helms et al., 2009)], since scan-rescan data were not available to estimate the intra-site variance. The validity of this particular measure of intra-site CoV is limited to areas with negligible physiological variations. Therefore, intra-site CoV estimates were only extracted in ROIs with homogeneous signal, i.e., the head of the caudate nucleus and the genu of the corpus callosum. The CoVs were averaged across sites for each volunteer.

The inter-site CoVs of GM probability maps derived from MT maps and T1w images were determined by calculating the mean and *SD* across sites within a GM mask. The GM mask included all voxels with a GM probability over 90%. To reduce potential bias against one of the two image types, the GM mask was derived from the corresponding type of map, i.e., either the T1w image or MT map. To formally assess whether the GM probability maps derived from MT maps had a lower inter-site CoV than the ones derived from the T1w images, a one-tailed paired *t*-test was performed with a significance threshold of *p* < 0.05.

## Results

**Figure 1:**
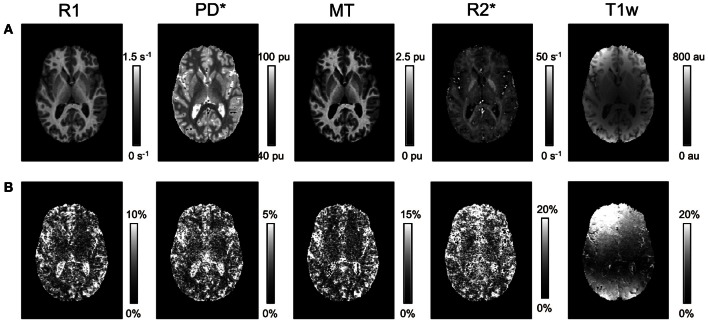
Axial slice through R1, PD^*^, MT, and R2^*^ parameter maps and T1w images for a single volunteer. (A) Mean and **(B)** inter-site coefficient of variation (CoV) across the three different sites.

Visual inspection of the parameter maps indicated a high quality of parameter maps with no gross artifacts (Figure 1). In contrast to the parameter maps, the T1w images showed an inhomogeneous and high inter-site CoV.

**Figure 2:**
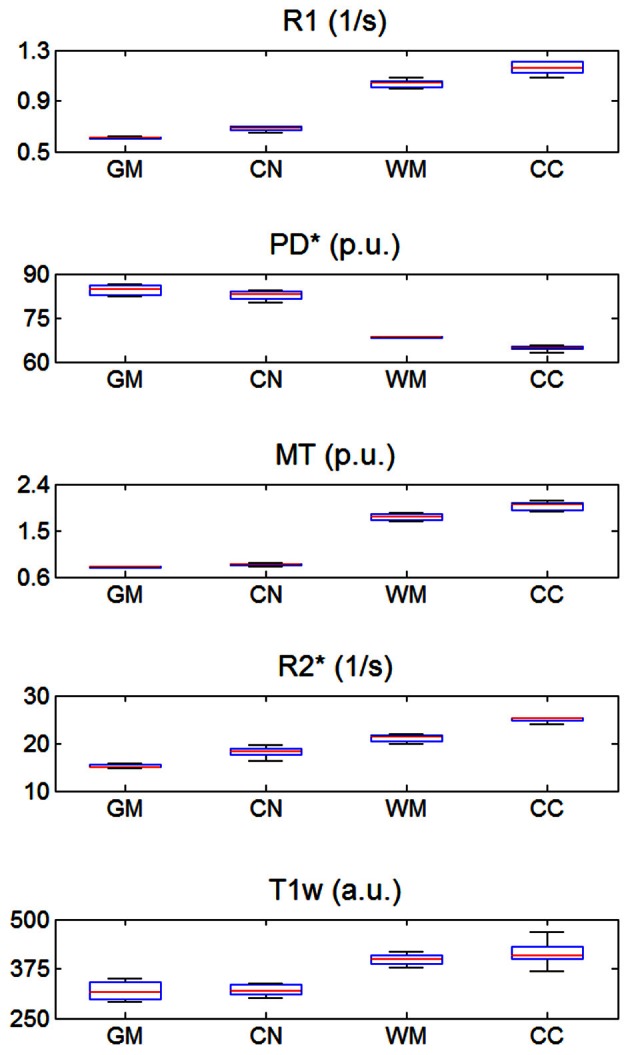
R1, PD^*^, MT, and R2^*^ parameter and T1w image intensity values averaged across the three different sites. Values were determined separately for gray matter (GM), head of the caudate nucleus (CN), white matter (WM) and genu of the corpus callosum (CC). The distribution across volunteers is depicted as a whisker plot: blue box = 25/75% percentile; red line, median, black whisker, most extreme data value excluding outliers; red cross, outlier (probability < 0.01 under assumption of normally distributed data).

The mean R1, PD^*^, MT and R2^*^ in the four ROIs (i.e., GM, WM, head of the caudate nucleus and genu of the corpus callosum) are presented in Table 1 and Figure 2.

**Table 1:** **Group mean and standard deviation of parameter values and T1w image intensity in different ROIs**.

**ROI**	**R1 (1/s)**	PD^*^ (p.u.)	**MT (p.u.)**	R2^*^ (1/s)	**T1w (a.u.)**
GM	0.609 ± 0.008	84.44 ± 1.87	0.794 ± 0.014	15.2 ± 0.4	319 ± 24
CN	0.683 ± 0.022	82.67 ± 1.64	0.836 ± 0.027	18.2 ± 1.2	321 ± 15
WM	1.036 ± 0.036	68.35 ± 0.06	1.764 ± 0.066	21.0 ± 0.8	398 ± 15
CC	1.158 ± 0.050	64.65 ± 0.86	1.978 ± 0.085	25.0 ± 0.5	414 ± 35

Mean ± standard deviation across five subjects. GM, gray matter; WM, white matter; CN, head of the caudate nucleus; CC, genu of the corpus callosum; R1, longitudinal relaxation rate;

PD* *, effective proton density; MT, magnetization transfer saturation*;

R2* *, effective transverse relaxation rate; T1w, signal intensity in T1w image*.

**Figure 3:**
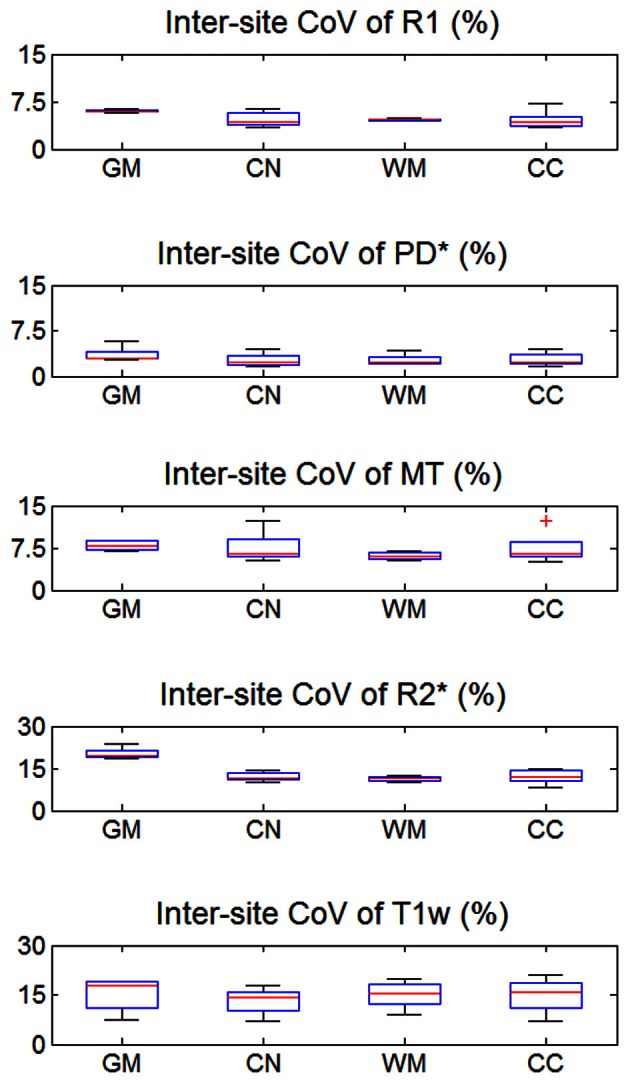
Inter-site coefficient of variation (CoV) of R1, PD^*^, MT, and R2^*^ parameter maps and T1w images intensity values. Values were determined separately for gray matter (GM), head of the caudate nucleus (CN), white matter (WM), and genu of the corpus callosum (CC). For explanation of the whisker plot, see Figure 2.

**Figure 4:**
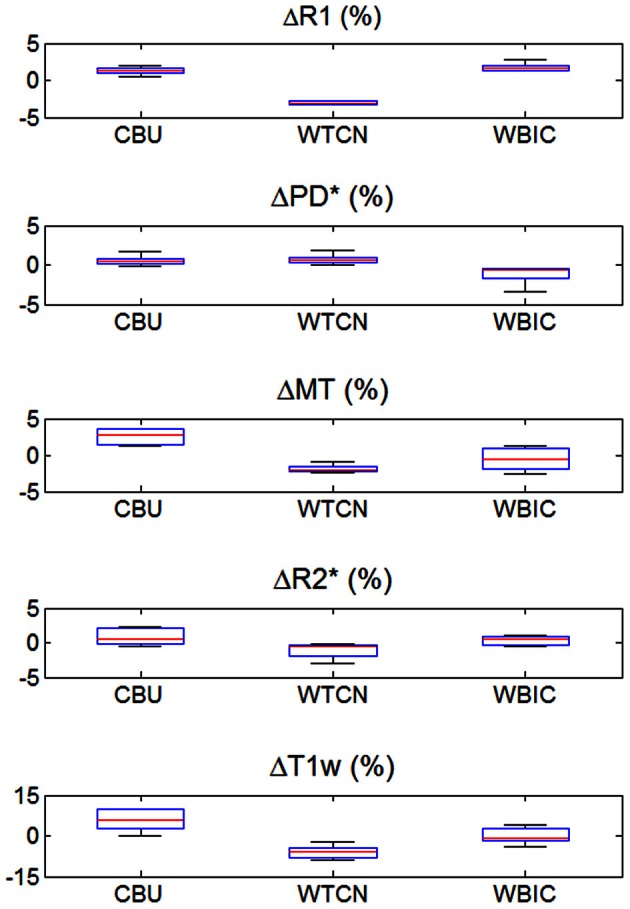
Inter-site bias of R1, PD^*^, MT, and R2^*^ parameter and T1w image intensity values in gray and white matter for the three different sites (except for PD^*^, which was assessed in gray matter only). To enhance visibility of any potential inter-site bias, the percent deviation from the mean across sites is shown and plotted for all volunteers. For description of the whisker plot, see Figure 2.

**Figure 5:**
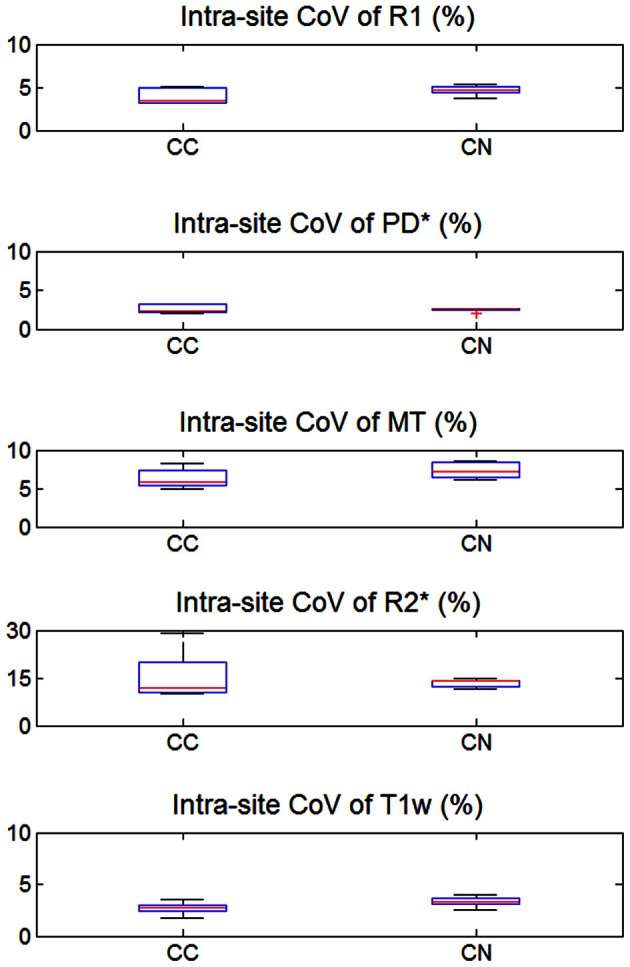
Intra-site coefficient of variation (CoV) of R1, PD^*^, MT, and R2^*^ parameter maps and T1w images intensity values. Values were determined separately for caudate nucleus (CN) and genu of the corpus callosum (CC). For explanation of the whisker plot, see Figure 2.

The inter-site CoV for R1, PD^*^ and MT parameters ranged from 2.7 to 7.9% (Table 2 and Figure 3). The inter-site CoV of R2^*^ ranged from 11.4% in WM to 20.3% in GM. The bias between the three different sites for the R1, PD^*^, MT and R2^*^ did not exceed 3.1% (Figure 4). The intra-site CoV for R1, PD^*^, MT and R2^*^ determined in the caudate head and genu of the corpus callosum were similar to the inter-site CoV, ranging from 2.4 to 15.7% (Table 3 and Figure 5).

**Table 2:** **Group mean and standard deviation of the *inter-site* coefficient of variation (CoV) in percent for the parameter values and T1w image intensity in different ROIs**.

**ROI**	**R1**	PD^*^	**MT**	R2^*^	**T1w**
GM	6.0 ± 0.2	3.6 ± 1.2	7.9 ± 0.8	20.3 ± 2.0	15.2 ± 5.2
CN	4.7 ± 1.2	2.7 ± 1.1	7.6 ± 2.7	12.0 ± 1.6	13.0 ± 4.1
WM	4.6 ± 0.1	2.7 ± 1.0	6.1 ± 0.6	11.4 ± 1.0	15.1 ± 4.2
CC	4.6 ± 1.5	2.7 ± 1.1	7.4 ± 2.8	12.1 ± 2.6	14.7 ± 5.5

For description of the used labels, see Table 1.

**Table 3:** **Group mean and standard deviation of the *intra-site* coefficient of variation (CoV) in percent for the parameter values and T1w image intensity in different ROIs**.

**ROI**	**R1**	PD *	**MT**	R2 *	**T1w**
CN	4.7 ± 0.6	2.4 ± 0.3	7.4 ± 1.1	13.3 ± 1.3	3.3 ± 0.5
CC	3.9 ± 0.9	2.6 ± 0.6	6.3 ± 1.3	15.7 ± 7.9	2.7 ± 0.7

For description of the used labels, see Table 1.

The T1w imaging showed a high inter-site CoV of ca. 15% but one of the smallest intra-site CoVs with ca. 3% (Tables 2, 3; Figures 3, 5). The inter-site CoV of the T1w images was significantly higher than that of the MT (*p* = 0.025, *F* = 12.1), R1 (*p* = 0.009, *F* = 22.5) and PD^*^ parameter maps (*p* = 0.003, *F* = 44.0). The R2^*^ maps did not show a significantly different inter-site CoV compared to the T1w images (*p* > 0.75, *F* = 0.1). In line with the higher inter-site CoV, an inter-site bias of up to 5.9% was measured for the T1w images, which was higher than for the quantitative parameters.

**Figure 6:**
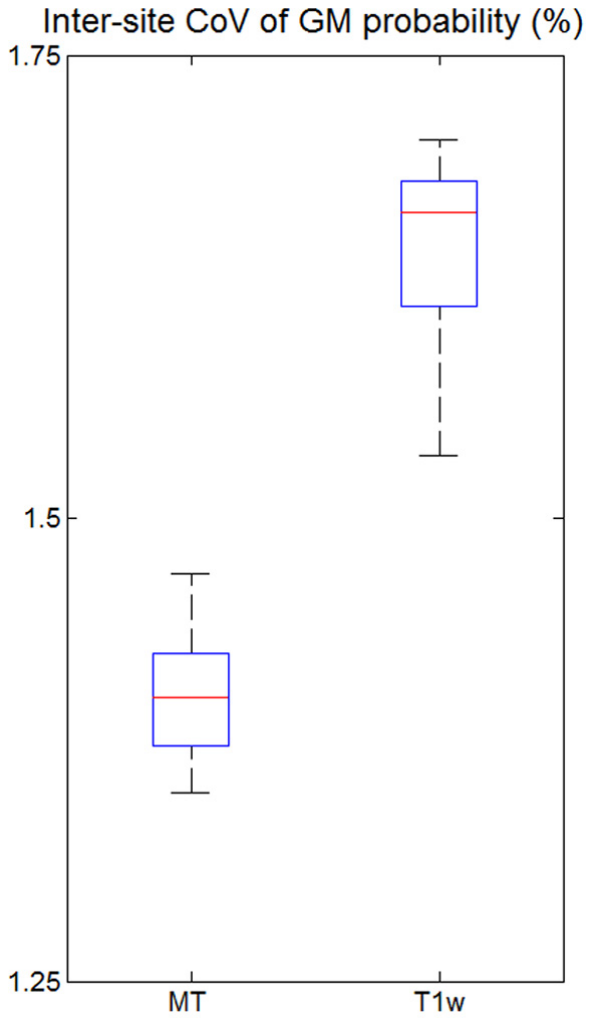
**Inter-site coefficient of variation (CoV) of gray matter (GM) probabilities for all five volunteers across the three different sites.** CoV was measured within the cortex as determined by thresholding the GM probability map (*p* > 0.9). For explanation of the whisker plot, see Figure 3.

The inter-site CoV of the GM probability maps (Figure 6) was 1.40% for the MT maps [with a 95% confidence interval of CI = (1.35, 1.46%)] and 1.64% for the T1w images [with CI = (1.56, 1.73%)]. Thus, the maps derived from MT maps had a ca. 14% higher inter-site reproducibility (*p* < 0.05).

## Discussion

This multi-center validation study demonstrated a high inter-site reproducibility of the MPM approach, which was significantly higher compared to conventional T1w imaging. The inter-site CoV was smaller than 8% for R1, PD^*^ and MT maps. R2^*^ maps exhibited a higher inter-site CoV of up to 20% (similar to standard T1w images) due to the rather short maximal echo times used to determine R2^*^. The inter-site bias (i.e., systematic offset) between the three different sites did not exceed 3.1% for any of the parameter values.

The inter-site CoV and inter-site bias for standard T1w images were significantly higher with 15% and 5.9%, respectively—as expected for a non quantitative imaging technique. GM probability maps based on MT parameter maps had a 14% higher inter-site reproducibility than maps based on T1w images.

### Comparison of parameter maps and T1w images

The quantitative parameter maps showed a higher inter-site reproducibility than the conventional T1w imaging normally used for neuoranatomical studies, since T1w imaging suffers from signal and contrast bias due to inhomogeneities in the RF transmit and receive field. At 3T RF transmit field inhomogeneities frequently exceed 20–30% of the nominal field (Lutti et al., 2010), causing significant signal and contrast bias (Thomas et al., 2005). The 32-channel receive head coil used in this study exhibits a highly inhomogeneous sensitivity profile varying by 200–300% across the brain (Wiggins et al., 2006). Both types of bias depend on the position and orientation of the head within the transmit and receive RF coils. We aimed at positioning the head as consistently as possible at the different sites by following an identical protocol. However, even relatively small deviations of 1–2 cm and 3–5° may have lead to significant signal variation in T1w images. This variation was most prominent in the superficial cortical areas close to the receiver coils reflected in a highly increased inter-site CoV (ca. 10-fold increased compared to the center; Figure 1).

Since MPM is designed to be insensitive to inhomogeneities in the RF transmit field and receive fields, the parameter maps showed a much lower CoV. Also the MPM maps did not show wide-spread spatial variation of inter-site CoV unlike the T1w images (Figure 1) or previous R1 mapping approaches that did not employ RF transmit field mapping [see e.g., Figure 3 in (Deoni et al., 2008)]. Also the inter-site bias of the quantitative parameter maps was between 2- to 10-fold smaller than of the T1w imaging (Figure 4).

### Gray matter probability maps

Due to their signal and contrast bias T1w image intensities are usually not directly used in analyses but used indirectly for morphometry (Ashburner et al., 2003). For example, voxel-based morphometry (VBM) segments the image into different tissue classes including GM and WM (Ashburner and Friston, 2005). After appropriate spatial normalization the local GM volume is compared voxel-wise between groups and volunteers (Ashburner, 2007). Obviously, accurate and precise segmentation is crucial but relies on T1w image intensities. Most segmentation methods account for bias in the signal intensity (Ashburner and Friston, 2005) but have to rely on assumptions about the smoothness of the bias field and cannot model or compensate for contrast bias. Helms et al. (2009) demonstrated improved segmentation of subcortical areas with MT maps compared to T1w images, since they have a high contrast-to-noise ratio and minimal bias. Similarly, we observed a 14% lower inter-site CoV in the GM probability maps based on the MT parameter maps instead of T1w images (Figure 6). Under the assumption of (independent and identically distributed) Gaussian noise the improved inter-site reproducibility would therefore reduce minimal group sizes by ca. 30% in multi-center VBM studies. Thus, segmentation and VBM results are expected to be not only improved for single site but also multi-center studies by the use of MT parameter maps.

### Variations in parameter maps and their causes

Some of the residual inter-site bias and CoV in the MPM were caused by the following mechanisms. The dual flip angle mapping approach used in MPM (Helms et al., 2008a) provides signal amplitude (proportional to PD) and R1 maps that need to be corrected for RF transmit and receive field inhomogeneities. We acquired highly accurate and precise RF transmit field maps with a total error of less than ca. 3% (Lutti et al., 2010, 2012) and corrected for imperfect RF spoiling, which leads to deviations from the Ernst signal equation underlying the R1 estimation (Preibisch and Deichmann, 2009; Yarnykh, 2010). Although small, this allows for errors of up to 6% in the R1 maps due to the quadratic dependence of the estimated R1 on the local flip angle.

The RF receive field effect on the PD map was minimized by image post-processing. Unified segmentation (Ashburner and Friston, 2005) was adapted to robustly determine and correct for the multiplicative receive coil sensitivity profile in the PD maps, similar to the previously developed UNICORT approach for correcting R1 maps (Weiskopf et al., 2011). However, if multiple and small receive coils are used, the spatial sensitivity profiles may become difficult to model due to the low spatial smoothness, potentially causing insufficient correction. We calibrated the mean WM PD value to 69 p.u. (Tofts, 2003), since the global offset in PD values cannot be accurately determined by the unified segmentation step. The calibration step may introduce a bias if pathologies or physiological changes affect the PD in the majority of WM.

The PD maps were estimated from averaged images acquired at different echo times, resulting in a mean echo time of 8.45 ms. Although the mean echo time was comparatively short, it introduced a certain R2^*^ image intensity weighting. To point out this potential bias, we called the estimated parameter effective proton density (PD^*^) in line with previous studies (Lin et al., 1997). Since the UNICORT post-processing step accounted for the global offset by calibrating the WM PD^*^ to 69 p.u., the mean overall reduction in signal and thus PD^*^ was corrected, which would otherwise spuriously reduce the PD^*^ by ca. 15% (assuming T2^*^ = 50 ms and TE = 8.45 ms). However, the PD^*^ estimates may be still locally biased in regions with high R2^*^ values due to high iron concentrations, such as parts of the basal ganglia and certain brainstem nuclei. To overcome this problem, the signal at TE = 0 may be extrapolated from the multi-echo dataset (Neeb et al., 2006). However, we decided not to apply this type of correction, since the signal extrapolation is potentially unstable and can increase the noise level significantly (Neeb et al., 2006).

The R2^*^ parameter maps yielded an approximately 100% higher CoV than the other parameter maps. Since the longest echo time acquired in the PDw FLASH multi-echo readout was 19.7 ms, the estimation of long T2^*^ (=1/R2^*^) found in GM, WM or CSF was complicated. The precision of the R2^*^ maps may be improved by increasing the maximal echo time, but this would also prolong the total acquisition time. Moreover, R2^*^ was estimated from the logarithm of the signal intensities (in the PDw images) at different echo times using a linear regression. Since the SNR of the different echoes varies, heteroscadisticity may have impacted the fit. The assumption of a mono-exponential signal decay described by R2^*^ may be violated in some brain areas [e.g., suffering from susceptibility artifacts (Neeb et al., 2006)], although the high spatial resolution of 1 mm reduced the effects of susceptibility artifacts on the signal decay due to a smaller within voxel spin phase coherence loss (Weiskopf et al., 2007).

The MT saturation parameter is a measure of the saturation due to the applied off-resonance RF pulse, which is highly correlated with macromolecular content and myelin density (Helms et al., 2008b; Draganski et al., 2011). The semi-quantitative MT saturation is largely insensitive to changes in the excitation flip angle, repetition time, RF field inhomogeneities or R1 of the tissue but depends on the power and frequency offset of the MT saturation pulse (Helms et al., 2008b, 2010). For example, a higher powered saturation pulse will lead to higher MT values. Thus, the MT saturation pulse has to be kept identical for different implementations if direct comparability is desired. Calibration of the MT effect or full MT quantification may ensure data comparability even when the saturation pulses differ (Sinclair et al., 2010; Volz et al., 2010).

RF transmitter instability and differences in the RF transmitter adjustment may have also contributed to the inter-site CoV. Non-linearities in the RF amplifier may have caused additional variation (Balezeau et al., 2011), since the FLASH images were acquired with significantly different RF transmit voltages to achieve the different desired excitation flip angles. These instabilities are known to cause variations in PD, R1 and MT maps (Stikov, 2010).

### Limitations and considerations

The MPM approach poses significant challenges to the MRI scanner hardware. The fast bipolar multi-echo readout demands high gradient performance. Multi-channel RF receive coils are required to achieve good parallel imaging capability with high SNR. The image reconstruction software and hardware must be capable of handling an approximately 20-times increased data rate compared to standard T1w imaging [e.g., 3D MDEFT, (Deichmann et al., 2004)].

This multi-center validation was based on data from five healthy volunteers and three different sites. We do not believe that the small sample size compared to conventional morphometric studies affected the primary results, since most of the quantitative comparisons were based on aggregate measures (e.g., means over ROIs) providing high statistical power due to their low noise.

We note that intra-site and inter-site CoVs were found largely comparable in this study. This observation supports that little variance is added by using different MR scanners. Thus, multi-centre studies can be conducted with high sensitivity. However, we note that the MPM performance may be degraded for non-compliant volunteers. For example, patients may have difficulties to minimize head motion or body motion, which can change the magnetic field in the head and affect data quality (Versluis et al., 2010). The MPM parameters are estimated from up to three acquired datasets and are sensitive to artifacts present in any of these runs. If relevant, these problems can be addressed by prospective motion correction (Maclaren et al., 2012) and phase navigator techniques (Versluis et al., 2010).

The three sites participating in this study used the same type of MRI scanner and RF coils. Thus, inter-site bias due RF coil and gradient coil differences is expected to be lower than for studies using different scanner types. Gradient non-linearities were not addressed in this study, since the MRI scanner type used exhibits a very high linearity (Mohammadi et al., 2012). They may be addressed by post-processing if necessary (Jovicich et al., 2006). To avoid bias, special attention needs to be paid to the exact implementation of the MR pulse sequences including RF pulses and RF spoiling (Yarnykh, 2010), which can differ between software versions and scanner manufacturers. Thus, we used custom-made optimized pulse sequences in this study.

The comparability of parameter maps or images is low if they are acquired at different field strengths (e.g., at 3T and the typical clinical field strength of 1.5T). R1 and R2^*^ significantly depend on the field strength (Oros-Peusquens et al., 2008), whereas MT and PD^*^ show a much smaller dependence. Thus, a direct comparison and pooling of data across field strengths are complicated but may not be impossible using appropriate relaxometry models (Rooney et al., 2007). Further studies are needed to develop and validate such an approach. While multi-center MPM studies at 1.5T should not pose particular problems, studies at higher fields, such as 7T, will require additional validation, since artifacts and bias are exacerbated (Lutti et al., 2012).

It is well-known that for large sample sizes even a small bias may become significant when classic frequentist statistics (testing against a null effect) are used. Quantitative MRI may help us with this fundamental issue, since the absolute quantitative values can be used to define minimal biological effect sizes, which are site independent and more generally applicable. Thus, it can inform statistical analyses, e.g., Bayesian approaches, which take into account prior information and minimal effect sizes. This helps researchers to avoid over-interpreting biologically implausible or clinically irrelevant effects including residual small inter-site bias.

We note that the reported inter-site bias should not be regarded a measure of absolute accuracy of the parameter maps. We did not aim to determine the accuracy with this study but only comparability across sites. Determining the accuracy would have required comparisons to gold standard measurements, which usually suffer from long acquisition times and low resolution (Weiskopf et al., 2011). The measured parameter values were relatively well in line with previous studies. For example, the R1 = 0.61/1.04 s^−1^ in GM/WM was similar to the R1 measured previously 0.63/1.19 s^−1^ (Wright et al., 2008). PD^*^ estimates of 84.4/82.7 p.u in GM/caudate nucleus were in line with previous studies reporting 81.1/81.5 p.u. (Volz et al., 2012) and 82.2/84.8 p.u. (Neeb et al., 2008) for the same structures. Measurements of R2^*^ in WM were also similar to previous studies [21.0 s^−1^ compared to 19.5 s^−1^ (Baudrexel et al., 2009) and 21.7 s^−1^ (Martin et al., 2008)]. However, R2^*^ in the head of the caudate appeared reduced compared to previous studies [18.2 s^−1^ compared to 26.2 s^−1^ (Martin et al., 2008)]. However, a comparison with literature values should be interpreted with care, since estimates vary considerably between studies. For example, even recent studies differ by more than 15% in estimated R1 values (Oros-Peusquens et al., 2008; Wright et al., 2008). This is probably caused by different resolutions, varying ROI definitions and varying biases present in the studies (Tofts, 2003).

We expect some bias in the PD^*^ and R2^*^ maps in areas suffering from susceptibility artifacts, since the mono-exponential decay model used here was violated in these areas and we only partially corrected the PD^*^ maps for R2^*^ related effects (e.g., increased inter-site CoV in the frontal cortex, Figure 1). Thus, e.g., differences in shim and head positioning may have affected R2^*^ values.

Signal variations within ROIs were used as proxies for intra-site CoV due to the absence of scan-rescan data. Although we chose small homogeneous ROIs (e.g., head of the caudate nucleus), it is likely that some variability in tissue architecture within the ROIs contributed to the intra-site CoV, spuriously increasing the estimates of the intra-site noise level. Furthermore, correlations between receive channels of the 32-channel RF receive head coil might have biased noise estimates (Hutton et al., 2012). This also explains why the intra-site CoV was paradoxically higher than the inter-site CoV for R2^*^ in the corpus callosum (Figures 3, 5).

The multi-echo FLASH based T1w imaging, which was compared to MPM here, is less widely used since the advent of MPRAGE and MDEFT based T1w imaging (Mugler and Brookeman, 1990; Deichmann et al., 2000, 2004), since they offer a higher GM/WM contrast-to-noise ratio in the cortex (Tardif et al., 2009). However, neither MPRAGE nor MDEFT completely corrects for RF field bias. Thus, we expect a similarly increased inter-site CoV for these MR pulse sequences as well.

## Conclusion

We have introduced and validated a MPM approach for multi-center studies at 3T. It provides high-resolution maps of R1, PD^*^, MT and R2^*^ and thus detailed insights into the brain microstructure in a clinically feasible acquisition time. An optimized multi-echo FLASH acquisition with low artifact level and high signal-to-noise ratio combined with correction for RF transmit and receive field inhomogeneities results in accurate and precise quantitative measures. The resulting high comparability of MPM data across sites and time points facilitates multi-center studies and federating large datasets.

### Conflict of interest statement

Edward T. Bullmore is employed half-time by GlaxoSmithKline (GSK) and half-time by the University of Cambridge; he holds stock in GSK. The other authors declare that the research was conducted in the absence of any commercial or financial relationships that could be construed as a potential conflict of interest.
